# The effect of gaze-contingent stimulus elimination on preference judgments

**DOI:** 10.3389/fpsyg.2015.01351

**Published:** 2015-09-09

**Authors:** Masahiro Morii, Takayuki Sakagami

**Affiliations:** ^1^Global Centre for Advanced Research on Logic and Sensibility, Keio UniversityTokyo, Japan; ^2^Department of Psychology, Keio UniversityTokyo, Japan

**Keywords:** eye movements, preference, gaze bias, decision-making, non-sensical figure

## Abstract

This study examined how stimulus elimination (SE) in a preference judgment task affects observers’ choices. Previous research suggests that biasing gaze toward one alternative can increase preference for it; this preference reciprocally promotes gaze bias. Shimojo et al. (2003) called this phenomenon the Gaze Cascade Effect. They showed that the likelihood that an observer’s gaze was directed toward their chosen alternative increased steadily until the moment of choosing. Therefore, we tested whether observers would prefer an alternative at which they had been gazing last if both alternatives were removed prior to the start of this rising gaze likelihood. To test this, we used a preference judgment task and controlled stimulus presentation based on gaze using an eye-tracking system. A pair of non-sensical figures was presented on the computer screen and both stimuli were eliminated while participants were still making their preference decision. The timing of the elimination differed between two experiments. In Experiment 1, after gazing at both stimuli one or more times, stimuli were removed when the participant’s gaze fell on one alternative, pre-selected as the target stimulus. There was no significant difference in the preference of the two alternatives. In Experiment 2, we did not predefine any target stimulus. After the participant gazed at both stimuli one or more times, both stimuli were eliminated when the participant next fixated on either. The likelihood of choosing the stimulus that was gazed at last (at the moment of elimination) was greater than chance. Results showed that controlling participants’ choices using gaze-contingent SE was impossible, but the different results between these two experiments suggest that participants decided which stimulus to choose during their first period of gazing at each alternative. Thus, we could predict participants’ choices by analyzing eye movement patterns at the moment of SE.

## Introduction

The production and measurement of preferences, defined as positive emotional valences for certain objects, has been of interest to psychology and related fields for decades. Specifically, the relationship between preferences and eye movements has been well studied. Eye movements can cause preferences, as in the case of the mere-exposure effect, famously demonstrated by Zajonc (1968), wherein looking at an object increases one’s preference for it. Eye movements can also identify preferences, for example, by using the preferential-looking method, where it is presumed that people tend to look more at stimuli that they prefer (Fantz, 1961, 1963, 1964).

Shimojo et al. (2003) analyzed eye movements during alternative forced-choice preference judgment tasks. Pairs of faces or pairs of figures made with Fourier descriptors were presented simultaneously on a computer screen and participants were asked to choose the more attractive stimulus. “Gaze likelihood” was defined as the proportion of gazing directed at their chosen stimulus. Gaze likelihood gradually increased from about 0.6 s before making a selection. This tendency was seen only for tasks in which participants were asked to choose the more attractive stimulus and not in tasks where individuals were asked to indicate the more unattractive or rounder face. The authors described this phenomenon as the “gaze cascade effect,” wherein the mere-exposure effect and preferential looking both operate to create a positive feedback loop; exposure increases preference and preference increases exposure. The authors proposed a dual-contribution model of preference formation with two kinds of input. One input is a cognitive assessment system in which participants compare with templates. The other input is orienting behavior, which contributes to preference decision-making by integrating preferential looking and mere-exposure in a positive feedback loop, leading to the conscious choice. The decision module would then be responsible for integrating information from these two inputs across time. A decision would be made when a threshold is reached.

Simion and Shimojo (2007) improved upon the procedure of Shimojo et al. (2003) by changing the duration of stimuli presentation; both stimuli were randomly removed after 800–5000 ms. Participants were asked to choose the more attractive among presented faces by pressing a key regardless of whether the stimuli had disappeared. Trials were classified as “early decisions” and “late decisions” based on whether the participant made their decision before or after the stimuli disappeared. In early decisions, participants were asked to confirm their decision by pressing the button again after stimulus offset. Gaze likelihood increased before the final button press (decision confirmation or decision, respectively) in both types of trials despite the absence of stimuli. This suggested that once a gaze cascade effect has started, visual orientation toward the location of the preferred image does not stop and acts independently of the presence of stimuli.

The gaze cascade effect proposed by Shimojo et al. (2003) has been criticized. Glaholt and Reingold (2009a) used images of photographic art and conducted two-alternative and eight-alternative forced-choice judgment tasks for preference and typicality. In the typicality judgment tasks, participants were asked to choose the most unusual stimulus. Results showed that gaze bias toward the chosen stimulus was observed not only in the preference judgment task but also in the typicality-judgment task. Accordingly, the authors suggested that gaze bias reflects a general characteristic of visual decision-making, not a mechanism specific to preference judgments. Similarly, other experiments have indicated that gaze bias can be nearly identical for non-preference decisions (Glaholt and Reingold, 2009b, 2011; Nittono and Wada, 2009; Schotter et al., 2010). One possible explanation for the discrepancy between studies is the use of different stimulus categories. Shimojo et al. (2003) originally used faces and figures made by Fourier descriptors. Other research groups have used pictures of natural scenes or geometric figures. Recent studies have demonstrated that stimulus type is an important factor in gaze cascade generation (Park et al., 2010; Liao et al., 2011). For example, Park et al. (2010) conducted a two-alternative preference judgment task using a novel stimulus and a repeatedly presented stimulus. Face stimuli elicited a familiarity preference, whereas natural scenes elicited a novelty preference. There was no strong bias for geometric stimuli. These results indicate that stimulus category might affect the gaze cascade phenomenon.

On the other hand, preference judgment is affected by some experimental manipulation. Shimojo et al. (2003) conducted gaze manipulation in Experiment 2 of their study based on the gaze cascade model. Two faces were repeatedly and alternately presented for 900 and 300 ms, respectively, before, the preference judgment. Faces alternated to the right and left in the gaze manipulation condition. Meanwhile in the control condition, both faces alternated in the same location. The preference bias for the alternative that was presented longer was seen in the manipulation condition, but not in the control condition. Accordingly, they concluded that gaze manipulation could directly influence the preference judgment.

The gaze cascade model predicts that participants will be gazing at their chosen stimulus at the time of judgment. Interestingly, this raises the question of whether participants simply prefer the stimulus at which they most recently gazed. Simion and Shimojo (2007) analyzed the relationship between preference judgments and stimulus elimination (SE) and found that participants were more likely to be gazing at their preferred stimulus immediately prior to SE. However, in their study, data were analyzed from multiple, random SE durations concurrently. Another study by Simion and Shimojo (2006) used a moving window to restrict participants’ visual field, but no research has controlled the timing of the SE. Accordingly, in this study, we manipulated the timing of SE and examined whether participants more often preferred the stimuli at which they were gazing at the time of SE. We predicted that if gazing behavior is important for preference judgments, the rate of choosing the stimulus that was most recently gazed at should be greater than chance.

## Experiment 1

In Experiment 1, we conducted a preference judgment task with non-sensical figures. The stimuli were eliminated when an individual gazed at a predefined target stimulus.

### Method

#### Participants

Eight Japanese adults (four women, four men; mean age 21.9 ± 1.2 years) participated in this experiment. All participants had normal or corrected-to-normal vision. All participants provided informed consent in accordance with a protocol approved by the local ethics committee of Keio University. They were individually tested and paid 870 Japanese yen for their participation.

#### Apparatus and Stimuli

**FIGURE 1:**
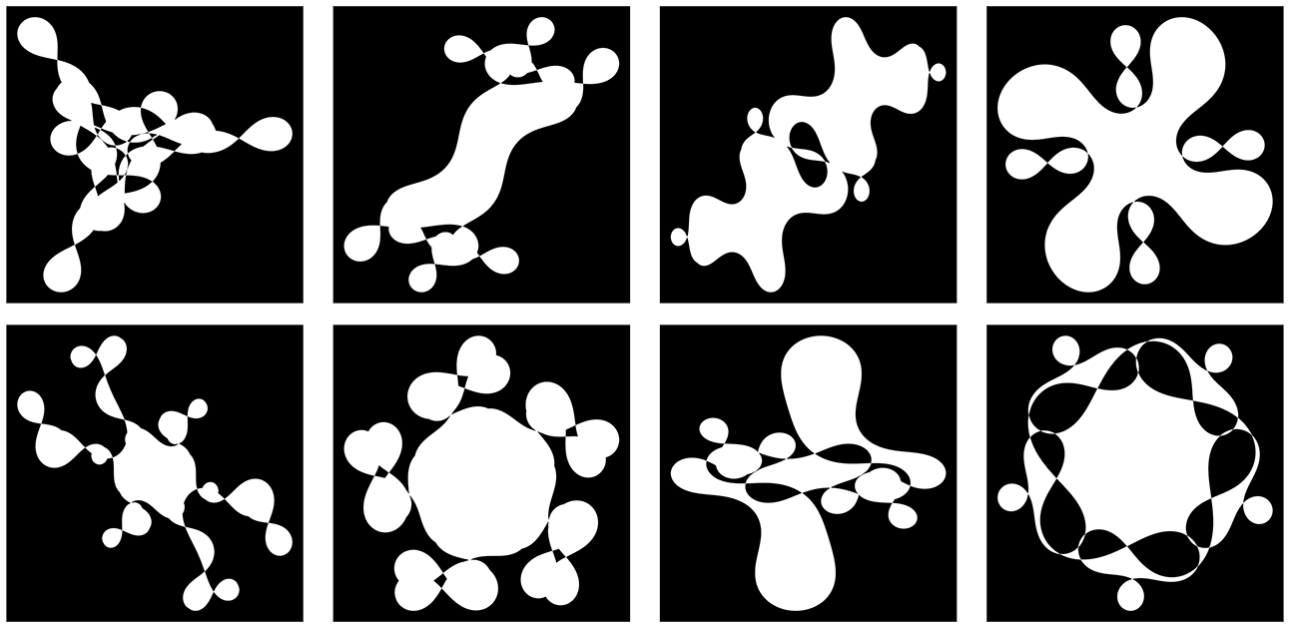
**Stimuli used in the experiment**.

Participants’ eye movements were recorded by the eye-tracking system, Eyelink 1000 (SR Research Ltd.). Stimuli were presented in the center of 23-inch display (Mitsubishi, model RDT234WX). The display was viewed from a distance of 75 cm and the head was stabilized. The display resolution was 1920 × 1080 pixels, and the visual angles were 37.5° horizontally and 21.6° vertically. Eight non-sensical figures were uniquely generated using a Fourier-descriptor algorithm based on Zahn and Roskies (1972). Stimuli were selected based on the pretest results. In the pretest, 135 figures were presented individually on the screen and 40 participants were asked to rate their preference for each figure on a scale from 0 (*very unattractive*) to 100 (*very attractive*). We selected the eight figures whose mean preference scores were closest to neutral with small SD. The actual preference scores ranged from 41.1 to 51.1 with SD less than 16.8. These figures are shown in **Figure 1**.

#### Procedure

After participants were informed of the experiment and procedures, they sat on a chair in front of a computer screen, and the calibration procedure of the eye-tracker was completed. The test phase consisted of 112 trials. All combinations of the eight figures were presented twice. One stimulus was predetermined to be the target stimulus. For example, there were two trials where figure A was presented on the right side and figure B on the left side; figure A was predetermined to be the target stimulus in one trial and figure B was predetermined to be the target stimulus in the other. The order of trials was counterbalanced across participants.

Fixation was defined as gazing at the stimulus area for more than 300 ms. In each trial, a fixation cross was presented and participants were asked to stare at it. When fixation on the cross was detected, it was removed and two figures were presented, one on each side of the screen. After fixation on both figures was detected, the figures were eliminated when the participant fixated again on the side that had contained the target stimulus. At the same time, an auditory tone was delivered to notify participants to be ready to respond. Participants were asked to inspect the stimulus pair freely until the stimuli were removed and to choose the more attractive stimulus by pressing a key with no time pressure. After making a choice, the next trial began with no inter-trial interval.

The target choice rate was defined as the proportion of choices of the target stimuli. The choice consistency rate was defined as the proportion of agreement between trial pairs with the same stimuli (but different target stimulus). Response latency was calculated as the time from SE to the key pressing.

### Results

#### Target Choice Rate and Choice Consistency Rate

The target choice rate, the choice consistency rate, and the mean response latency for each participant are shown in **Table 1**. The average target choice rate was 0.491 (*SD* = 0.036), and the average choice consistency rate was 0.821 (*SD* = 0.078). An independent samples *t*-test of arcsine-transformed choice rates indicated that choice rate did not differ from chance [*t*(7) = -0.71, n.s., Cohen’s *d* = 0.25]. Meanwhile an arcsine-transformed choice consistency rates were also higher than chance [*t*(7) = 9.80, *p* < 0.001, Cohen’s *d* = 3.47].

**Table 1:** Choice rate, consistency rate, and mean response latency in Experiment 1.

Participant	Target choice rate	Consistency rate	Choice latency (ms)
A	0.446	0.821	748.2
B	0.518	0.893	892.1
C	0.491	0.804	685.1
D	0.491	0.732	868.5
E	0.482	0.929	700.7
F	0.563	0.696	681.4
G	0.473	0.839	764.5
H	0.464	0.857	1149.2
**Average (*SD*)**	0.491 (0.036)	0.821 (0.078)	811.2 (158.2)

#### Gaze Likelihood

**FIGURE 2:**
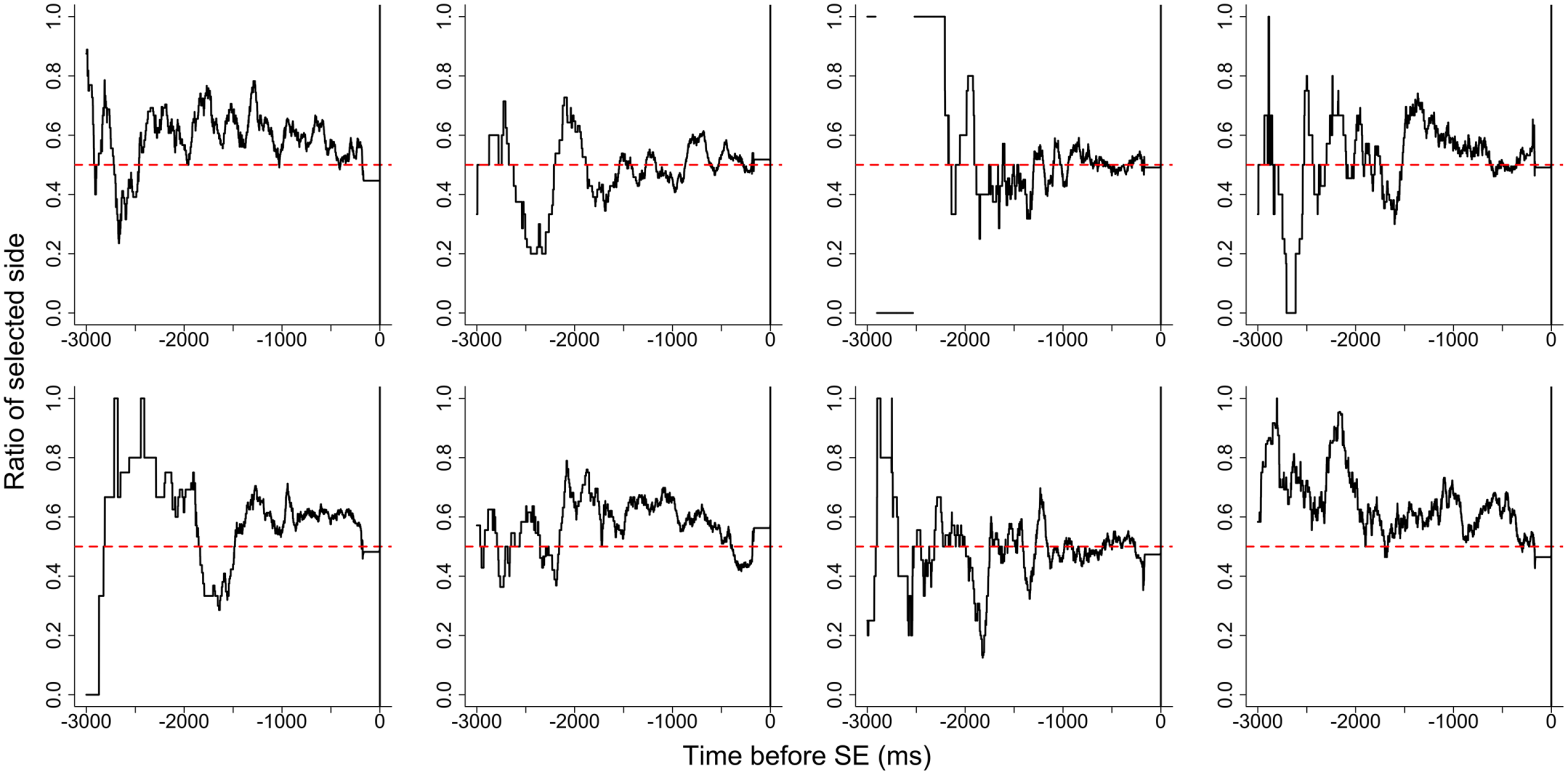
**Gaze likelihood before stimulus elimination (SE) for each participant in Experiment 1**.

**FIGURE 3:**
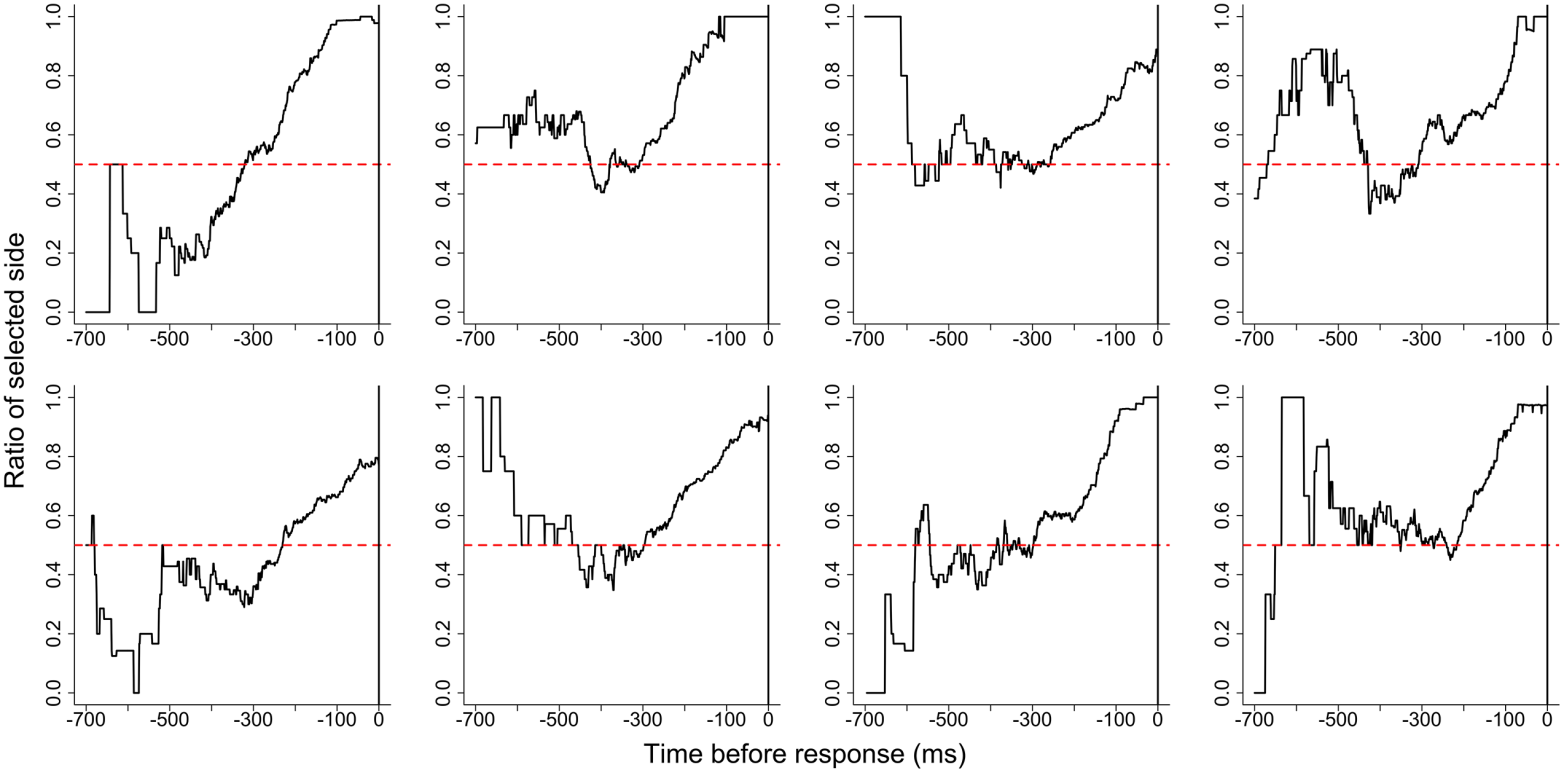
**Gaze likelihood after stimulus elimination for each participant in Experiment 1**.

Gaze likelihood before SE for each participant is shown in **Figure 2**. The gaze likelihood was around chance levels (0.500) and gaze bias was not observed before the SE. **Figure 3** shows gaze likelihood from the SE to response. Participant gaze gradually shifted toward the chosen alternative about 500 ms before the response despite the absence of stimuli.

### Discussion

In Experiment 1, we examined whether gaze-contingent SE biased choices in a preference judgment task. Despite SE being contingent upon gazing at the target stimulus, there was no difference in the rate of choosing target and non-target stimuli (i.e., there was no choice bias). The results of the gaze likelihood analyses showed that gaze bias was not observed before the SE but was observed before responses. This result is consistent with the results of Simion and Shimojo (2007). This means that participants shifted their gaze prior to key pressing regardless of stimuli presentation.

One possible cause for the lack of any observed SE effect may be the nature of the stimuli. We used eight non-sensical figures for which participants were expected to have no strong preferences. Interestingly, however, choice consistency rates were high, suggesting that participants had reliable preferences for certain stimuli. Strong inherent preferences could have masked the smaller effect of our SE manipulation. Another possible cause for these results relates to the procedures. In this experiment, stimuli sometimes were not eliminated smoothly because participants had immediately decided which stimulus to choose and gazed only at that stimulus. To account for these problems in Experiment 1, we improved the procedure in Experiment 2.

## Experiment 2

In Experiment 2, the timing of the SE was varied. We did not predetermine the target stimulus. We used another type of non-sensical figure and conducted a pretest using different participants in order to select experimental stimuli.

### Method

#### Participants

Eight different Japanese adults (four women, four men; mean age 23.8 ± 3.0 years) participated in this experiment. All participants had normal or corrected-to-normal vision. They were tested after providing informed consent and paid 870 Japanese yen for their participation.

#### Apparatus and Stimuli

The apparatus and stimuli were the same as in Experiment 1.

#### Procedure

The procedure was identical to Experiment 1, except for the timing of the SE. No target stimulus was predetermined in Experiment 2. Fixation was defined as gazing at a stimulus’ area for more than 300 ms. After fixation on both figures was detected, the figures were eliminated and an auditory tone was delivered when the participant was gazing again at either stimuli. A test phase was consisted of 112 trials. As before, all combinations of the eight figures were presented twice. The order of trials was counterbalanced across participants. Concordance rate was defined as the proportion of trials in which the target of fixation immediately prior to the SE and the preference choice were consistent.

### Results

#### Concordance Rate and Choice Consistency Rate

The results of Experiment 2 are shown in **Table 2**. The average concordance rate was 0.575 (*SD* = 0.068), and the average choice consistency rate was 0.806 (*SD* = 0.101). An independent samples *t*-test of the arcsine-transformed concordance rates indicated that the rate of choosing the stimulus that was gazed at immediately prior to SE (e.g., the third fixation) was significantly greater than chance [*t*(7) = 3.10, *p* < 0.05, Cohen’s *d* = 1.01]. An arcsine-transformed choice consistency rates were also higher than chance [*t*(7) = 7.28, *p* < 0.001, Cohen’s *d* = 2.57].

**Table 2:** Concordance rate, consistency rate, and response latency in Experiment 2.

Participant	Concordance rate	Consistency rate	Choice latency (ms)
I	0.652	0.804	645.9
J	0.500	0.696	1808.3
K	0.554	0.893	566.4
L	0.563	0.875	708.9
M	0.509	0.946	1440.6
N	0.518	0.804	1223.8
O	0.634	0.643	926.3
P	0.670	0.786	656.8
**Average (*SD*)**	0.575 (0.068)	0.806 (0.101)	997.1 (450.2)

#### Gaze Likelihood

**FIGURE 4:**
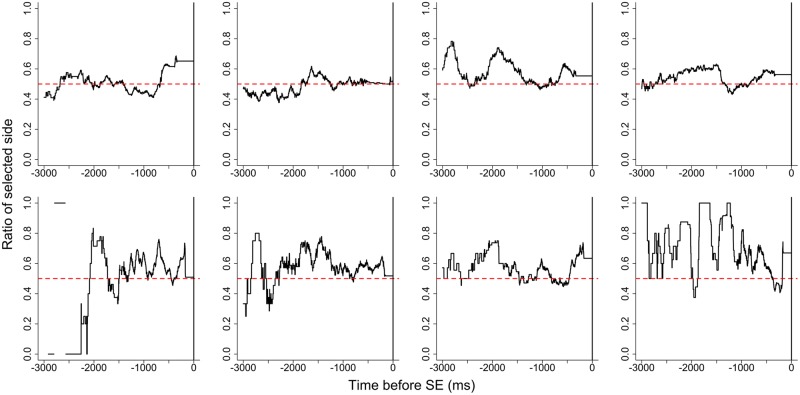
**Gaze likelihood before stimulus elimination for each participant in Experiment 2**.

**FIGURE 5:**
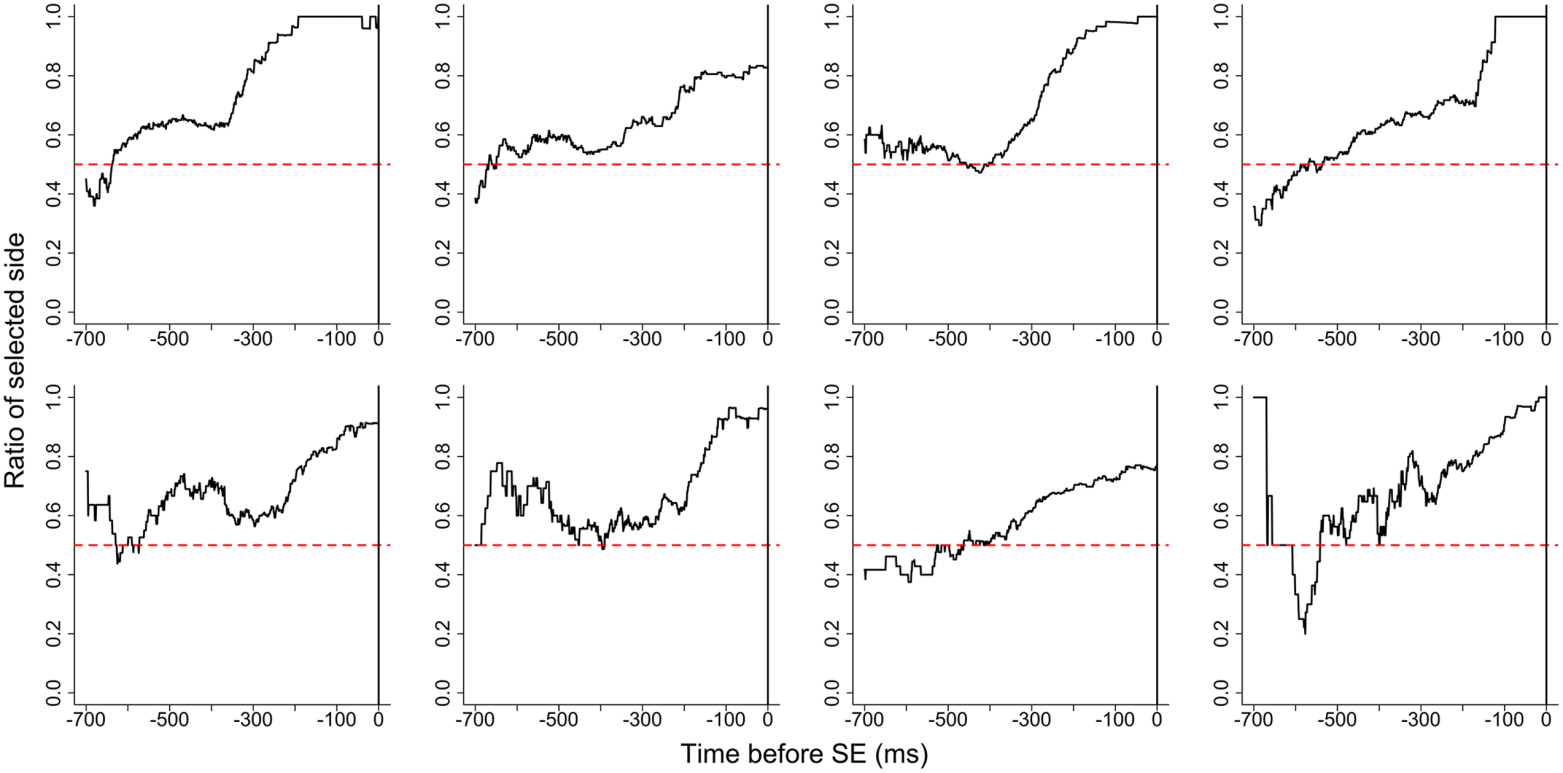
**Gaze likelihood after stimulus elimination for each participant in Experiment 2**.

The gaze likelihood before the SE is shown in **Figure 4**, and the gaze likelihood following the SE is shown in **Figure 5**. The gaze likelihood before the SE was greater than chance, but the patterns of increase were different across participants. For some participants, the gaze likelihood increased in the 1000–2000 ms prior to the SE. From the SE to the response, gaze likelihood gradually increased about 400–500 ms before responding, similar to Experiment 1.

#### Comparison with Experiment 1

**FIGURE 6:**
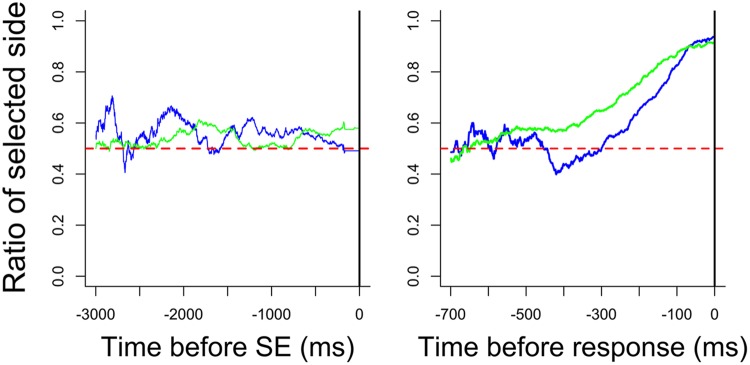
**Mean gaze likelihoods in each experiment**. Blue and green lines represent the mean likelihood in Experiments 1 and 2, respectively.

An unpaired *t*-test was performed after arcsine-transformation in order to compare the results of both experiments. The concordance rate in Experiment 2 was higher than the target choice rate in Experiment 1 [*t*(14) = 3.08, *p* < 0.01, Cohen’s *d* = 1.54]. Meanwhile the choice consistency rate in Experiment 2 was not significantly different from that in Experiment 1 [*t*(14) = 0.26, n.s., Cohen’s *d* = 0.13]. The mean gaze likelihoods in both experiments are shown in **Figure 6**. The gaze likelihoods fluctuated up and down above chance before SE in both experiments, and the likelihood was higher in Experiment 1 than it was in Experiment 2 at the moment of SE. The gaze likelihood before the response gradually increased about 400 ms before responding in both experiments. The likelihood in Experiment 2 was generally higher than it was in Experiment 1.

### Discussion

In Experiment 2, we did not predetermine the target stimulus and examined whether one’s fixation immediately prior to SE was related to actual choices. The results showed that the rate of choosing this pre-SE fixation stimulus was greater than chance, indicating that pre-SE fixation could predict preference judgments. However, these results require further evaluation before concluding that the timing of SE caused this phenomenon. As in Experiment 1, the choice consistency rate in Experiment 2 was high, suggesting that after fixating on the two stimuli, participants may have already judged which stimulus they preferred and then fixated again more often on the stimulus they would ultimately choose. Furthermore, in most trials participants gazed in the order of right–left–right or left–right–left. It might be interpreted from this that the effect of gazing upon a stimulus when it is removed is not important, but rather that the stimulus gazed upon first is most important for determining preference. In order to resolve this issue, future studies will need to eliminate stimuli at other times (e.g., after the fourth or fifth fixation).

Gaze likelihood analyses indicated that an increasing pattern was not observed among the participants before the SE. As in Experiment 1, gaze likelihood did increase between 300 and 600 ms before responding, despite the absence of stimuli. This result is consistent with the results of Simion and Shimojo (2007) in which the gaze likelihood increased before the final button press despite the absence of stimuli. This supports the notion that gaze bias is independent of the presentation of stimuli.

## General Discussion

The aim of this research was to examine the effect of removing stimuli in a preference judgment task. Specifically, we explored whether preferences were biased toward the image that was the target of gaze fixation immediately prior to SE. We observed no choice bias in Experiment 1, where one stimulus was predetermined to be the target triggering SE. Choice bias was observed in Experiment 2, where no target stimulus was predetermined and stimuli were eliminated only when the participant had gazed at both stimuli and was gazing again at either of the stimuli. These results indicate that we could predict a participant’s preference based on which stimulus they gazed at after having gazed at both.

According to our gaze likelihood analyses, participants gazed at both stimuli equally before the SE in Experiment 1. In Experiment 2, however, gaze was directed more toward the chosen alternative prior to the SE. Additionally, gaze gradually shifted toward the chosen alternative after the SE until making a response in both experiments. Simion and Shimojo (2007) explained that the gaze bias before the response was caused by the orienting behavior that acts independently of the presence of visual stimuli and they concluded that the gaze cascade effect is a preference-specific phenomenon. However, the findings are explainable by the idea that the gaze bias was not caused by the feedback loop of mere-exposure and preferential looking suggested by the gaze cascade model, but rather this gaze bias may reflect preparing for the response. As noted above, some previous studies showed that the time-series variation of gaze likelihood before the response for non-preferential tasks does not differ from that for preferential tasks (Glaholt and Reingold, 2009b, 2011; Nittono and Wada, 2009; Schotter et al., 2010). These phenomena, also interpreted as gaze bias, reflect a general characteristic of visual decision-making rather than a preference-specific mechanism. Further studies are needed to confirm that such gaze bias is a general characteristic.

This research has some limitations. There is no way of knowing when and how preferences were judged, and they may have occurred well before SE. To solve this problem, other types of procedures would be effective. One method would be to make the stimulus visible only when gaze is fixed at the area of the stimulus. Another method would be to use a greater number of stimuli, instead of repeating the same stimuli. If the preference-choice bias observed in our study persists under these conditions, the possibility of predicting and controlling one’s preferences based on their final fixation prior to stimulus removal would be more reliable.

## Author Contributions

Both authors conceived the design of experiment. MM conducted the experiments, analyzed the data, and wrote the first draft of the manuscript. Both authors contributed equally to later drafts approved the final manuscript, and are jointly responsible for the accuracy and integrity of the work.

## Conflict of Interest Statement

The authors declare that the research was conducted in the absence of any commercial or financial relationships that could be construed as a potential conflict of interest.
